# Probing *in vivo* RNA Structure With Optimized DMS-MaPseq in Rice

**DOI:** 10.3389/fpls.2022.869267

**Published:** 2022-03-31

**Authors:** Qiongli Jin, Linqi Zhang, Saiyan Hu, Guangbo Wei, Zhiye Wang

**Affiliations:** State Key Laboratory of Plant Physiology and Biochemistry, College of Life Sciences, Zhejiang University, Hangzhou, China

**Keywords:** RNA structure, DMS-MaPseq, rice, phosphate homeostasis, miRNA

## Abstract

RNA transcripts form various secondary and tertiary structures that have a wide range of regulatory functions. Several methods have been developed to profile *in vivo* RNA secondary structure in prokaryotes and eukaryotes. These methods, such as dimethyl sulfate (DMS) mutational profiling with high-throughput sequencing (DMS-MaPseq), couple small chemical-mediated RNA modifications with next-generation sequencing. DMS-MaPseq, a powerful method for genome-wide and target-specific RNA secondary structure profiling, has been applied in yeast, mammals, *Drosophila*, and *Arabidopsis thaliana*, but not in crops. Here, we used DMS-MaPseq to conduct a target-specific and genome-wide profile of *in vivo* RNA secondary structure in rice (*Oryza sativa*). The DMS treatment conditions were optimized for rice leaf and root tissues. To increase the sequencing depth and coverage of low-abundance transcripts in genome-wide DMS-MaPseq, we used streptavidin-biotin depletion to reduce the abundance of highly expressed chloroplast transcripts during library construction. The resulting target-specific and genome-wide rice DMS-MaPseq data were of high quality and reproducibility. Furthermore, we used DMS-MaPseq to profile the *in vivo* RNA secondary structure of an OsmiR399 target region located at 5′UTR of *OsPHO2*, which participates in rice phosphate homeostasis. An unfolded RNA structure downstream of miRNA target site was observed in predicted *in vivo* RNA secondary structure, reminiscence of the TAM (Target Adjacent nucleotide Motif) involved in mRNA structure-mediated regulation in miRNA cleavage. Our study optimized DMS-MaPseq for probing *in vivo* RNA secondary structure in rice, facilitating the study of RNA structure-mediated regulations in crops.

## Introduction

RNA transcripts form diverse secondary and tertiary structures *via* intra- and inter-molecular RNA base pairing. In living cells, RNA folding is dynamic and largely dependent on the cellular context. Growing evidence has shown that *in vivo* RNA structure has critical functions and plays important regulatory roles in numerous biological processes, such as precursor messenger RNA (mRNA) processing, RNA stability, RNA trafficking, translation and phase separation in prokaryotes and eukaryotes (Bevilacqua et al., 2016; Vandivier et al., 2016; Yang et al., 2018; Zhu et al., 2021). In plants, many studies have revealed the versatility of *in vivo* RNA structures, involving in splicing, polyadenylation, translation, microRNA (miRNA) biogenesis, miRNA-mediated RNA silencing, mRNA long-distance transport, RNA N^6^-methyladenosine (m^6^A) modification, plant development, ambient stress responses, and other processes (Ding et al., 2014; Kwok et al., 2015a; Hawkes et al., 2016; Zhang et al., 2016, 2019; Foley et al., 2017; Cho et al., 2018; Deng et al., 2018; Su et al., 2018; Wang et al., 2018; Wu et al., 2019; Chung et al., 2020; Kramer et al., 2020; Tack et al., 2020; Yang et al., 2020a,b, 2021; Gawronski et al., 2021; Liu et al., 2021; Reis et al., 2021).

Several methods coupling small chemical-mediated RNA modification with high-throughput sequencing have been developed to precisely profile complicated *in vivo* RNA secondary structures at a single nucleotide resolution (Spitale et al., 2013, 2015; Ding et al., 2014; Rouskin et al., 2014; Wu and Bartel, 2017; Mustoe et al., 2018; Weng et al., 2020). Many probing chemicals are applied, such as selective 2′-hydroxyl acylation analyzed by primer extension (SHAPE) reagents, DMS, N_3_-kethoxal, glyoxals and 1-ethyl-3-(3-dimethylaminopropyl)carbodiimide (Ding et al., 2014; Rouskin et al., 2014; Spitale et al., 2015; Mitchell et al., 2018, 2019; Weng et al., 2020). DMS is one of the most widely used chemicals for *in vivo* RNA secondary structure probing due to its high reactivity and strong ability to penetrate cells (Kubota et al., 2015; Zhu et al., 2021). DMS modifies the Watson-Crick face of unpaired adenosine (A) and cytosine (C) to N^1^-methyladenosine (m^1^A) and N^3^-methylcytidine (m^3^C; Wells et al., 2000). The DMS-elicited modifications block traditional reverse transcription (RT), generating RT stops at complementary DNA (cDNA).

In so-called RT stop methods, 3′ ends of cDNA mapped to the transcriptome indicate the single-stranded regions of RNAs (Kwok et al., 2015b). Two early genome-wide *in vivo* RNA structure probing methods, Structure-seq (Ding et al., 2014) and DMS-seq (Rouskin et al., 2014), were developed based on the RT stop method and successfully applied in bacteria, yeast and plants (Ding et al., 2014; Rouskin et al., 2014; Burkhardt et al., 2017; Ritchey et al., 2017; Su et al., 2018; Zhang et al., 2018; Tack et al., 2020). However, RT stop-based methods require appropriate DMS treatment with single-hit kinetics conditions, otherwise over-reaction by DMS causes highly skewed distribution of RT stops near the primer-binding sites. In addition, 3′ end RNA structure information is difficult to obtain due to short sequencing reads at the 3′ end of RNA. Another limitation is that degraded RNAs introduce false positive signals in the RT stop methods (Wu and Bartel, 2017; Zubradt et al., 2017; Yang et al., 2020a).

Alternatively, a new strategy called mutational mapping (MaP) was developed (Siegfried et al., 2014; Zubradt et al., 2017). Instead of using RT stops, the DMS-MaPseq method transfers DMS modifications on RNAs to cDNA mutations *via* a special RT enzyme called thermostable group II intron reverse transcriptase (TGIRT; Mohr et al., 2013; Zubradt et al., 2017). TGIRT mostly generates mismatch mutations on cDNA, with few insertions or deletions (indels). Therefore, TGIRT is preferred over other MaP methods that use an RT with SuperScript II (SSII) plus Mn^2+^, as this generates high numbers of indels (Zubradt et al., 2017). The high-fidelity of TGIRT endows the DMS-MaPseq method with single-nucleotide resolution and a high signal-to-noise ratio (Zubradt et al., 2017). Furthermore, DMS-MaPseq can be used to specifically investigate the RNA structure of low-abundance transcripts and isoforms (Zubradt et al., 2017; Guenther et al., 2018; Wang et al., 2018). Recently, two algorithms, DREEM (Detection of RNA folding Ensembles using Expectation-Maximization; Tomezsko et al., 2020) and DRACO (Deconvolution of RNA Alternative COnformations; Morandi et al., 2021), were developed to identify coexisting alternative RNA conformations of the same transcripts based on DMS-MaPseq data.

In plants, DMS-MaPseq was first used in a study of miRNA biogenesis. Wang et al. (2018) used target-specific DMS-MaPseq to profile the secondary structure of primary miRNAs (pri-miRNAs) between the wild-type (WT) and the mutant of the SWI/SNF chromatin remodeling factor CHROMATIN REMODELING 2 (CHR2)/BRAHMA in *Arabidopsis thaliana*. It demonstrated that CHR2 remodels the secondary structure of pri-miRNAs to impede miRNA biogenesis (Wang et al., 2018). Later, the authors optimized genome-wide DMS-MaPseq for Arabidopsis materials (Wang et al., 2019). In addition, a recent study performed DMS-MaPseq, as well as SHAPE-seq, to query the RNA structure of chloroplast transcripts and revealed RNA structure-mediated translational regulation of *psbA* and other plastid genes with weak Shine-Dalgarno sequences (Gawronski et al., 2021). However, the use of DMS-MaPseq in plants has been limited to Arabidopsis.

**Figure 1:**
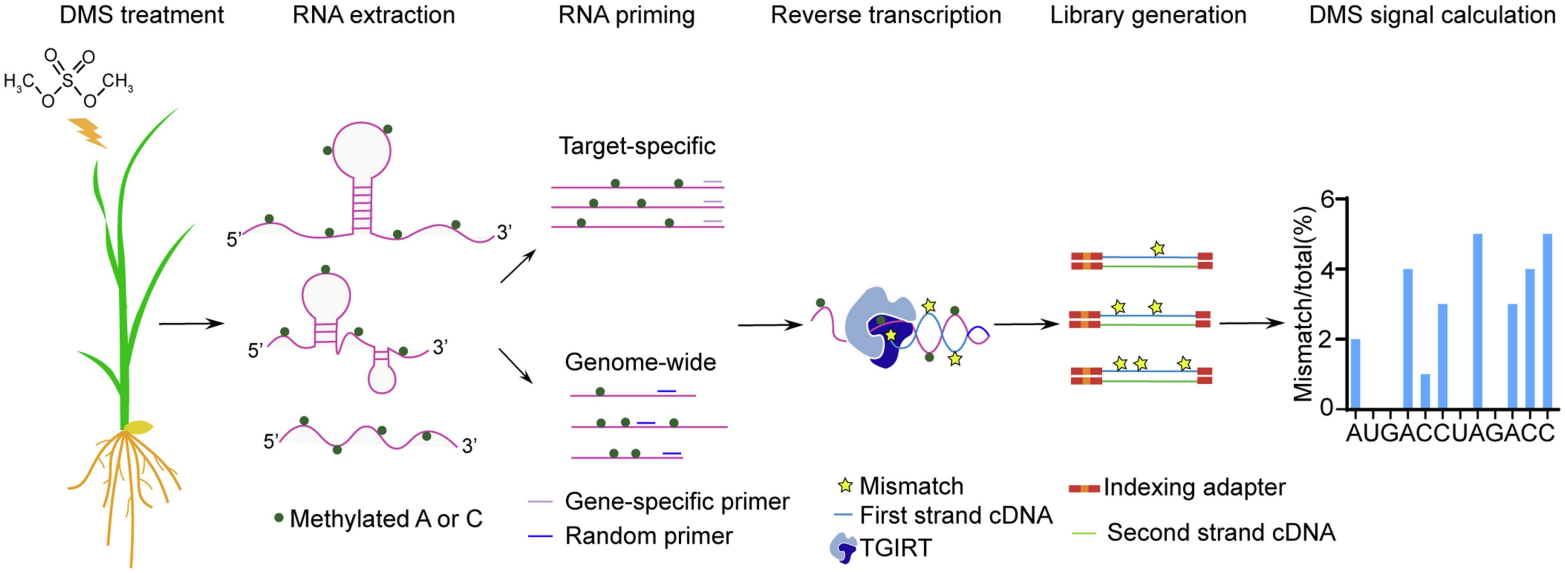
Workflow of optimized target-specific and genome-wide DMS-MaPseq in rice.

In this study, we optimized DMS-MaPseq for profiling the *in vivo* RNA secondary structure in rice (*Oryza sativa*; Figure 1). We optimized DMS treatment conditions for rice leaves and roots. Then we assessed the quality and reproducibility of our target-specific and genome-wide DMS-MaPseq data. To improve the sequencing depth and coverage for genome-wide DMS-MaPseq, we adopted a streptavidin-biotin depletion approach to reduce the abundance of highly expressed chloroplast transcripts during library construct. Then, we validated the feasibility of our rice DMS-MaPseq data for *in vivo* RNA secondary structure prediction. Finally, we used our RNA structure data to model the *in vivo* RNA secondary structure of a key regulator of rice phosphate (Pi) homeostasis. Altogether, the optimized DMS-MaPseq for rice could facilitate the study of RNA structure-mediated biological functions in crops.

## Materials and Methods

### Plant Material and Growth Conditions

Rice (*Oryza sativa* ssp. *Japonica* Nipponbare) seedlings were grown in a hydroponic nutrient system at 30°C under a 12 h light-12 h dark cycle. Three-week-old seedlings were harvested for DMS treatment.

### DMS Treatment

DMS treatment was performed as described (Wang et al., 2018, 2019). Three-week-old rice seedlings grown in hydroponic solution were collected and immersed in 20 ml of 1× DMS reaction buffer (40 mM HEPES pH 7.5, 100 mM KCl and 0.5 mM MgCl_2_). Then, 200, 300, 400, 500, or 600 μl of DMS (MACKLIN, Cat#: D824267) was added to the reaction buffer to give a final concentration of 1, 1.5, 2, 2.5, and 3%, respectively. For DMS treatment control, leaves of 3-week-old Arabidopsis grown on the soils were collected and treated with 1% DMS. For the no DMS control, the same volume of DEPC-treated water was added into the reaction buffer. DMS treatment was performed for 15 min at 30°C with shaking at 250 rpm or under vacuum (approximately 12 psi) at room temperature without shaking. After the DMS treatment, 6 ml of β-mercaptoethanol (Sigma, Cat#: M6250) was added to a final concentration of 23% and incubated under vacuum for 5 min to quench the DMS reaction. Then, the samples were washed three times with DEPC-treated water, frozen in liquid nitrogen, and ground to fine powder.

### RNA Extraction

The powdered rice materials (0.1 g) were mixed with 1 ml of Trizol reagent (invitrogen, Cat#: 15596018) and total RNA was extracted following the manufacturer’s protocol. Denatured agarose gel electrophoresis was used to validate the integrity of total RNA.

### Primer Extension Assays

The DMS treatment was validated by a primer extension assay as previously described (Wang et al., 2018, 2019) with some modifications. For each sample, 3 μg of TURBO DNase (Thermo Fisher, Cat#: AM2238) treated total RNA was mixed with 0.25 μl of 2 μM biotinylated 18S rRNA RT primer (Supplementary Table 1). The mixture was precipitated by ethanol and re-suspended in a 12 μl of RNase-free H_2_O. The solution was heated to 75°C for 3 min and put on ice for at least 1 min. Then, 4 μl of 5× First Strand buffer (250 mM Tris–HCl pH 8.3, 375 mM KCl, 15 mM MgCl_2_), 1 μl of 0.1 M DTT, 1 μl of 10 mM dNTPs and 1 μl of SUPERase-In RNase Inhibitor (Thermo Fisher, Cat#: AM2694) were added. The mixture was heated at 35°C for 15 min, and 1 μl of SuperScript III reverse transcriptase (Thermo Fisher, Cat#: 18080093) was added. The reaction was incubated at 55°C for 1 h, then inactivated by heating at 70°C for 15 min. Following phenol-chloroform extraction and ethanol precipitation, the cDNA was size-fractionated on a 10% urea-polyacrylamide gel. Next, the cDNA was transferred to a positively charged nylon membrane (GE Healthcare, Cat#: RPN303B) *via* a semi-dry blotter (Bio-rad). Immobilized cDNA was detected following the procedure of the chemiluminescent nucleic acid detection module kit (Thermo Fisher, Cat#: 89880) and the signal was collected with iBright1500 (Thermo Fisher).

### Target-Specific DMS-MaPseq

Target-specific DMS-MaPseq was performed as described (Zubradt et al., 2017; Wang et al., 2018) with some modifications. Following DNase treatment, 3 μg of non-DMS-treated sample or 6 μg of DMS-treated sample was mixed with 0.5 μl of 10 μM gene-specific RT primers mixture (Supplementary Table 1). The mixture was precipitated and re-suspended in a 10 μl of Tris-KCl solution (50 mM KCl and 10 mM Tris-HCl, pH 7.5). The solution was heated at 75°C for 3 min, followed by incubation at 57°C for 15 min. Then, 4 μl of 5× First-Strand buffer, 1 μl of 0.1 M DTT, 1 μl of SUPERase-In RNase inhibitor (Thermo Fisher), 1 μl of RNase-free H_2_O and 1 μl of TGIRT-III (InGex, Cat#: TGIRT50) were added to the solution. After incubation at room temperature for 30 min, 2 μl of 10 mM dNTPs was added and reverse transcription was conducted at 60°C for 2.5 h. Then, 2 μl of 2.5 M NaOH was added to stop the reaction and decay the RNA. The mixture was incubated at 95°C for 3 min and neutralized by adding HCl. Next, the cDNA was purified with RNAClean XP beads (Beckman). Then, the targets were amplified with KOD-FX hot-start DNA polymerase (Toyobo) using gene-specific primers (Supplementary Table 1). PCR products were gel purified and normalized according to band intensity. The library was constructed with NEBNext Ultra II DNA Library Prep Kit for Illumina (NEB). The libraries were quantified using Agilent TapeStation before sequencing by 2 × 250 bp paired-end reads on the Illumina Novaseq 6000 at Novogene.

### *In vitro* Transcription of Biotinylated Anti-chloroplast RNA Probes

Chloroplast genes were amplified using listed primers containing T7 promoter sequence (Supplementary Table 1) and cloned into TA/Blunt-Zero vector (Vazyme). The resulting plasmids were used as the PCR template to amplify DNA templates for *in vitro* transcription. Then, PCR products were gel purified with a PCR purification kit (Vazyme). The reaction mixture including 250 ng of purified DNA templates, 1 μl of 100 mM ATP, 1 μl of 100 mM GTP, 1 μl of 100 mM CTP, 0.4 μl of 100 mM UTP, 0.8 μl of 50 mM biotin-16-UTP (Lucigen), 1.5 μl of T7 RNA Polymerase Mix (NEB) and 1 μl of SUPERase-In RNase inhibitor (Thermo Fisher) was incubated at 37°C overnight. Then, TURBO DNase (Thermo Fisher) was added to digest the DNA template at 37°C for 1 h. Finally, RNA probes were purified by RNAClean XP beads (Beckman) and mixed with a final concentration of 160 ng/μl anti-*PSBA* probes, 70 ng/μl anti-*RBCL* probes, 15 ng/μl anti-*PSAB* probes, 15 ng/μl anti-*PSAA* probes, 10 ng/μl anti-*PSBC* probes, 10 ng/μl anti-*PSBB* probes, and 10 ng/μl anti-*PSBD* probes.

### Chloroplast RNA Depletion

Total RNA was extracted from DMS-treated rice leaves and was treated with TURBO DNase (Thermo Fisher). One microgram of DNase-treated total RNA was mixed with 0, 0.25 or 0.5 μl anti-chloroplast RNA Probe mixture in a 20 μl hybridization reaction (50 mM Tris-HCl pH 7.5, 100 mM NaCl). The mixture was put in a thermocycler at 68°C for 5 min, then ramped down by −0.1°C/s to 22°C, and finally held at 22°C for 5 min. Then, 100 μl of Dynabeads MyOne Streptavidin C1 (Thermo Fisher, Cat#: 65001) was washed and re-suspended in 40 μl of 2× binding and washing buffer according to the manufacturer’s manual. Biotinylated probe-target hybrids were immobilized by streptavidin beads twice. The depleted RNA solution was precipitated by ethanol and re-suspended in 10 μl of RNase-free H_2_O. Then, 0.75 μl of random primer and 0.75 μl of 10 mM dNTP were added to 10 μl of RNA. The mixture was heated to 65°C for 5 min and incubated on ice for at least 1 min. cDNA was synthesized by adding 4 μl of 5× First-strand buffer, 0.75 μl of 0.1 M DTT, 0.75 μl of SUPERase-In RNase inhibitor (Thermo Fisher) and 0.5 μl of TGIRT (InGex), and 2.5 μl of RNase-free H_2_O. The reverse transcription was performed at 25°C for 10 min, then 42°C for 30 min, then 60°C for 1.5 h. Next, 2 μl of 2.5 M NaOH was added to stop the reaction and decaying RNA. The expression of chloroplast RNAs were measured by RT-PCR. The intensity of PCR products was quantified by ImageJ. The primers used for RT-PCR were listed in Supplementary Table 1.

### Genome-Wide DMS-MaPseq

Genome-wide DMS-MaPseq was performed as described (Wang et al., 2019) with some modifications. DMS-MaPseq library was constructed using Illumina TruSeq® Stranded Total RNA Sample Prep Plant kit (Illumina) and TGIRT enzyme (InGex). Following DNase treatment and RNA purification using RNasey Mini Kit (QIAGEN), 1 μg DMS treated or untreated total RNA was mixed with 0.25 μl of home-made biotinylated anti-chloroplast RNA Probe mixtures and rRNA removal Probes (Ribo-zero rRNA removal kit for plant, illumina). The mixture was incubated in the thermocycler at 68°C for 5 min, and ramped down by −0.1°C/s to 22°C, and finally held at 22°C for 5 min. Then, probe-target hybrids were removed following the kit’s protocol. DMS-MaPseq libraries were constructed as described (Wang et al., 2019). The libraries were sequencing by 2 × 150 nt paired-end reads on Novaseq 6000 at Novogene.

### Sequencing Alignment and Analysis

Data analysis for target-specific or genome-wide DMS-MaPseq was performed as described (Wang et al., 2019; Tomezsko et al., 2020). Briefly, after read quality filtering with TrimGalore,^1^ clean reads were mapped to the reference genome using TopHat^2^ with parameter settings: --library-type fr-firststrand --no-novel-juncs -N 15 --read-gap-length 10 --read-edit-dist 15 --max-insertion-length 5 --max-deletion-length 5 -g 3. In that, the 10% mismatch tolerance setting (-N 15 for 150 nt sequencing reads) is based on a previous study (Zubradt et al., 2017). Next, uniquely mapped reads were extracted from the bam file using the Linux command grep with NH:I:1 tag. After discarding mismatches located within 3 nt of an indel, mutations and sequencing depth were counted from the uniquely mapped bam file. The DMS mutation signal was calculated for each adenine (A) and cytosine (C) nucleotide as mismatch/sequencing depth. The used Python scripts are described in the previous study (Wang et al., 2019).

### *In vivo* RNA Structure Prediction

Based on the DMS mutation signal, the secondary structures were modeled by RNAStructure (Reuter and Mathews, 2010).^3^ DMS signals were color coded on structure models using VARNA.^4^

### Graph Drawing

Graphs with dot plots (individual data points) were drawn using GraphPad Prism 8^5^, R,^6^ or Adobe Illustrator CC.

### Accession Codes

The GEO accession number of the DMS-MaPseq data in this study is GSE197245.

## Results

### Optimization of DMS Treatment Conditions for Rice

**Figure 2:**
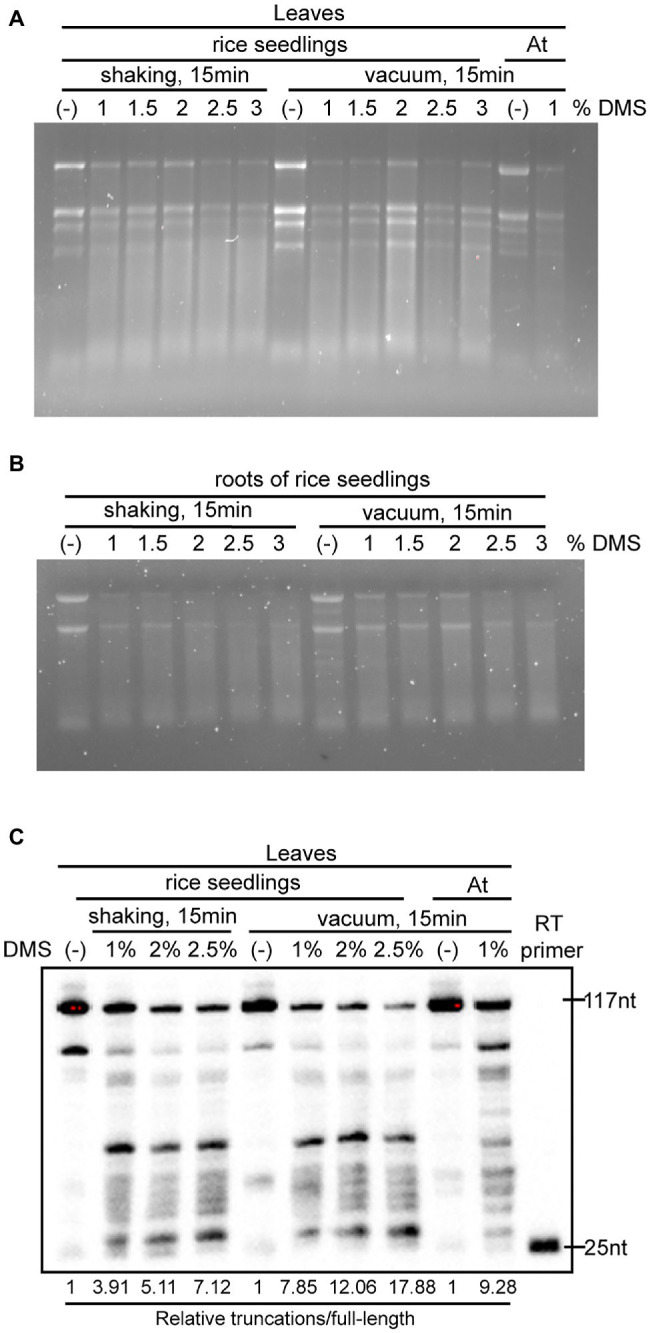
Optimization of DMS treatment for different rice tissues. **(A,B)** Images of denaturing gel electrophoresis showed the integrity of total RNA from untreated and DMS-treated rice leaves **(A)** and roots **(B)**. **(C)** 18S rRNA primer-extension assays validated the extent of DMS modification on RNA under various DMS concentrations and incubation conditions. The extent of DMS modification is indicated by the ratio of the signal intensity of total truncations to full-length cDNA (Relative truncations/full length). The ratio of truncations/full length was normalized to that of untreated samples, where the number was arbitrarily set to 1.

DMS-MaPseq requires that DMS penetrates into cells to modify RNA *in vivo*. However, rice absorbs a lot of silicon from the soil and deposits it in the leaves, stem, and husks to form silica bodies (Ma and Yamaji, 2006). These silica bodies serve as a physiological barrier, which not only resists pathogen infection and lodging but also hinders chemical penetration (Tamai and Ma, 2003; Ma et al., 2006). To optimize the DMS treatment conditions for rice tissues, we treated 3-week-old rice leaves with varying DMS concentrations (1, 1.5, 2, 2.5, and 3%, *v*/*v*) and different incubation conditions (30°C with shaking at 250 rpm for 15 min, or with vacuum for 15 min). We also treated 3-week-old Arabidopsis leaves as a reference. As seen in the Arabidopsis sample, total RNAs of DMS-treated rice samples were slightly degraded while untreated RNA was intact (Figure 2A), consistent with previous finding that high DMS concentrations cause RNA degradation (Wang et al., 2019). Moreover, the extent of decay was correlated with the DMS concentration, in which RNA treated with higher concentrations of DMS appeared more severely decayed. This indicated DMS modifications on the RNAs. We also found that shaking- and vacuum-treated samples exhibited similar RNA degradation patterns in denaturing agarose gel electrophoresis (Figure 2A).

Unlike Arabidopsis that is a taproot system dominated by the primary root, rice has a fibrous root system dominated by vast crown roots and lateral roots. Rice roots contain multiple layers of cortex cells from several to more than 10 layers, impeding the penetration of DMS into inner cells (Henry et al., 2017). To assess the efficacy of the DMS treatment conditions on rice roots, we treated roots of 3-week-old hydroponically cultured rice plants with different DMS concentrations (from 1 to 3%, *v*/*v*) and incubation conditions (shaking or vacuum for 15 min). Consistent with the leaf results, total RNAs of root samples were modified under our DMS treatment conditions (Figure 2B). Of note, the treatments with greater than 2% DMS caused very severe RNA decay (Figure 2B), which may compromise subsequent profiling of *in vivo* RNA structure. This suggested the optimal DMS treatment varies for different tissues. The tested DMS treatments for rice roots are much harsher than the optimized condition for Arabidopsis roots (0.75% DMS and 1 min incubation; Tack et al., 2020), indicating DMS is more difficult to penetrate rice roots than Arabidopsis roots.

To further measure the extent of DMS modification of RNA, we performed primer extension assays for 18S ribosome RNA (rRNA) in untreated and DMS-treated leaf samples. The primer extension assay was modified from a previously published protocol (Wang et al., 2018), with some modifications. Briefly, we used a 5′ end biotinylated 18S rRNA-specific primer and chemiluminescent detection, instead of ^32^P-labeled primer and autoradiography. Compared to the untreated samples, DMS treatments led to less full-length cDNA and more truncations (Figure 2C), implying DMS modifications of RNA. Consistent with this, image quantification showed that higher DMS concentrations caused an increase in the ratios of truncations-to-full-length cDNA (Figure 2C). Of note, the vacuum-treated samples had a moderately higher truncations-to-full-length cDNA ratio than the corresponding shaking-treated samples, suggesting that DMS penetrates into plant cells more efficiently under vacuum than shaking (Figure 2C).

Altogether, these results suggested that our DMS treatment conditions efficiently modified *in vivo* RNA. Higher DMS concentration treatment results in more DMS modifications on RNA, but also causes greater RNA decay that deteriorates the quality of DMS-MaPseq library (Figure 2). We recommended using 1–2% DMS with shaking or vacuum for rice leaves and 1% DMS with shaking for rice roots.

### Assessment of Target-Specific DMS-MaPseq Data

**Figure 3:**
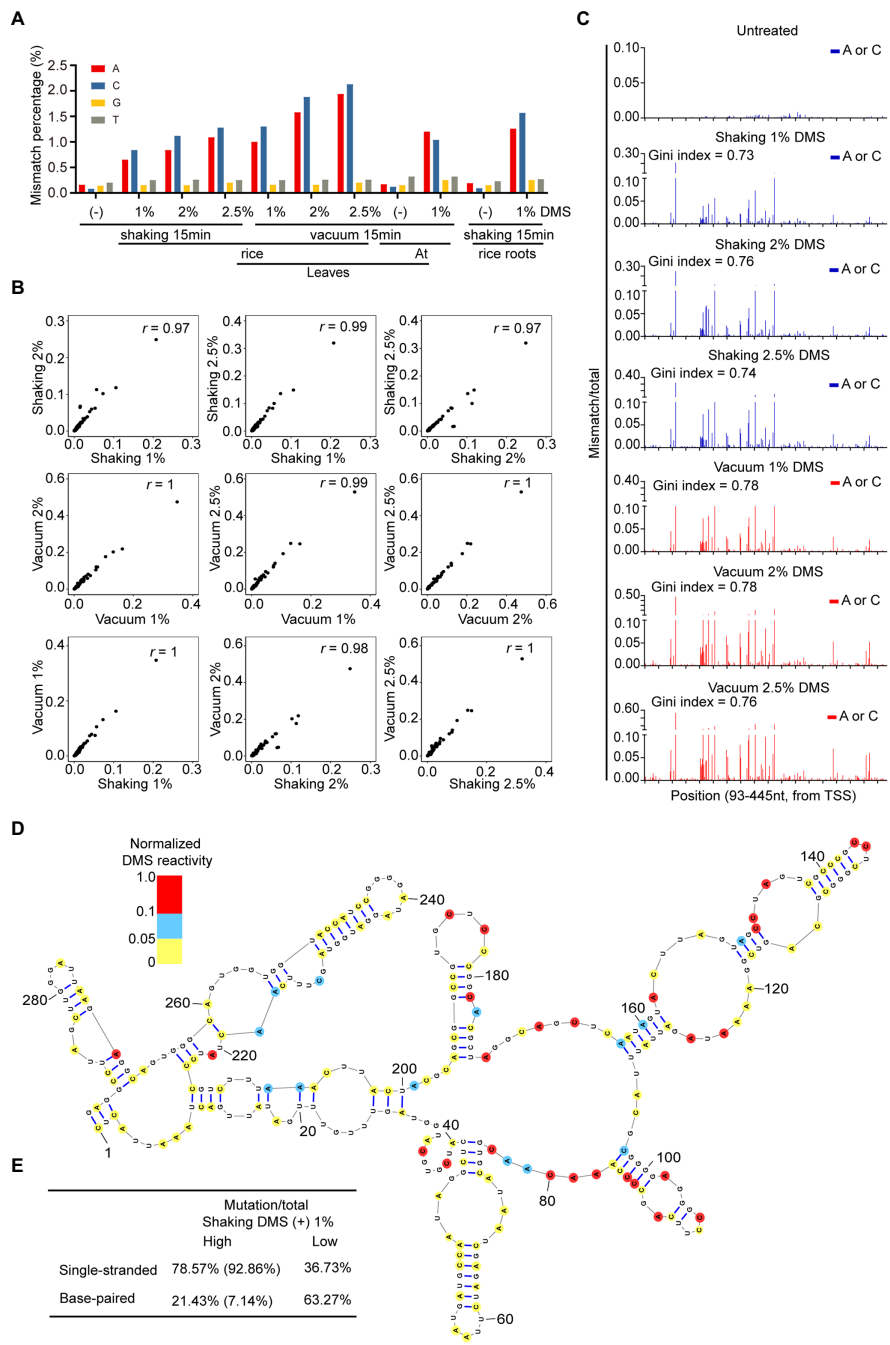
High-quality of target-specific DMS-MaPseq data in rice. **(A)** Total mismatch percentage on each nucleotide in untreated and DMS-treated 18S rRNA region (93-445nt). **(B)** High correlation of DMS-MaPseq signal for A and C nucleotides in tested 18S rRNA (93–445 nt) region under varying DMS concentrations and incubation conditions. Pearson’s *r* values are shown. The data in *X*-axis and *Y*-axis were mismatch/total mutation rate in corresponding DMS treatment conditions. **(C)** Ratiometric DMS signals of each A and C were plotted along 18S rRNA sequence. Similar Gini index values indicated the high similarity in distribution pattern of DMS mutation signals in different DMS-treated samples. TSS, Transcriptional start site. **(D)** Nucleotides 98–390 of the phylogenetic rice 18S rRNA structure was color-coded according to the DMS mutation signal from DMS-MaPseq. **(E)** High correlation between DMS mutation signal and 18S rRNA phylogenetic structure. The ratiometric DMS signal per position normalized to the highest mismatch/total in the displayed region, which was set arbitrarily to 1.0. In tested 18S rRNA region (from 98 to 390 nt), 78.57% (true positive) of As and Cs that showed high DMS mutation signal (defined as normalized DMS activity ≥ 0.1) in our DMS-MaPseq data corresponded to single-stranded regions in the phylogenetic structure, whereas 63.27% (true negative) of As and Cs that showed low DMS mutation signal (defined as normalized DMS activity ≤ 0.05) in our DMS-MaPseq data corresponded to base-paired regions in the phylogenetic structure. Of the 21.43% (false positive) nucleotides (defined as normalized DMS activity ≥ 0.01) that were annotated as base-paired in phylogenetic structure, 66.67% nucleotides were positioned either at the end of a helix or proximal to a bulge or loop, which were known be flexible. Corrected for these positions, the values in parentheses showed higher true positive and lower false positive percentages.

DMS alkylates A and C located in single-stranded regions. Then, DMS lesions on RNA are decoded as mismatches on cDNA through TGIRT (Zubradt et al., 2017). To assess the quality of DMS-MaPseq data with different DMS treatment conditions, we amplified a region of 18S rRNA (93–445 nt) from untreated and treated samples, followed by target-specific DMS-MaPseq and bioinformatic analysis. We examined the enrichment of DMS-induced mismatches on nucleotides. Compared to the untreated sample, the percentage of mismatches located at A and C, but not guanosine (G) and thymidine (T), was dramatically increased in DMS-treated samples (Figure 3A). This indicates high signal-to-noise ratios in our DMS-MaPseq data. Furthermore, higher DMS concentrations led to more mismatches in A and C, indicating a dosage-dependent effect of DMS on RNA modification (Figure 3A). In addition, vacuum treatment generated more mismatches than shaking, consistent with the results of the primer extension assay (Figures 2C, 3A). We also found root sample with 1% DMS and 15 min shaking treatment exhibited a similar mismatch percentage on each nucleotide with leaf samples of Arabidopsis and rice, suggesting that 1% DMS with 15 min shaking is optimized to rice root tissue (Figure 3A).

Then, to assess the fidelity of DMS-MaPseq data from the different DMS treatments, we conducted a correlation analysis among samples treated with different DMS concentrations. The results revealed excellent correlation in the DMS mutation signals (the ratios of mismatches to total reads) among the 1, 2, and 2.5% DMS-treated samples (Figure 3B). We also compared DMS mutation signals between shaking and vacuum treatment and observed high correlation (Figure 3B). In addition, the different DMS treatments resulted in highly similar DMS mutation signals distribution patterns along the tested region of 18S rRNA (Figure 3C). These results showed high fidelity of the *in vivo* RNA structure data generated by various treatments, suggesting that the DMS-MaPseq method can tolerate high DMS concentrations.

To test whether our DMS-MaPseq data accurately profiled *in vivo* RNA secondary structure, we mapped our DMS mutation signals of 1% DMS treated leaf sample to the evolutionally conserved 18S rRNA secondary structure (Ding et al., 2014). The results revealed high consistency between our DMS mutation signals and the well-known 18S RNA secondary structure, indicating our DMS-MaPseq data are consistent with the *in vivo* RNA structure (Figures 3D,E).

In summary, these results demonstrated the high quality of our rice DMS-MaPseq data.

### Optimization of Genome-Wide DMS-MaPseq for Rice Materials

Genome-wide DMS-MaPseq requires extensive sequencing depth and coverage to generate reliable global RNA structure information (Zubradt et al., 2017; Wang et al., 2019). However, for species with large genome sizes, such as crops, in-depth sequencing would be costly and a computational burden. One way to address this issue is to reduce the amount of highly expressed transcripts (Wang et al., 2019).

**Figure 4:**
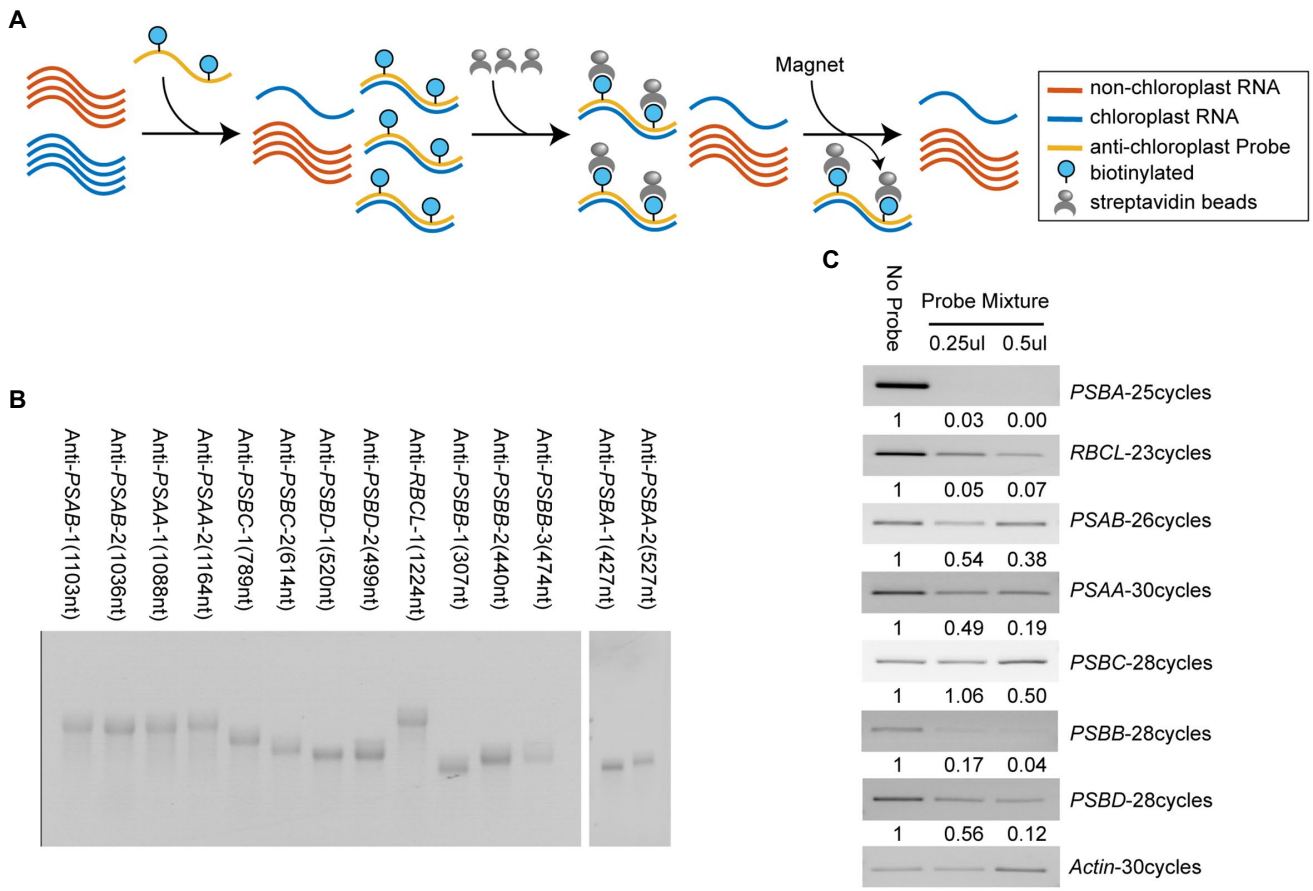
Specific depletion of highly-expressed chloroplast transcripts. **(A)** Workflow of the streptavidin-biotin depletion approach for reducing abundance of highly-expressed chloroplast RNAs. **(B)** Images of denatured gel electrophoresis showed home-made biotinylated RNA probes were high purity. **(C)** RT-PCR results showed efficient depletion of specific chloroplast transcripts using antisense biotinylated RNA probes.

Since chloroplast transcripts are highly expressed and account for almost half of the total coding RNA in plant leaves, we adopted a streptavidin-biotin depletion approach to decrease the abundance of chloroplast transcripts from total RNA (Figure 4A). Several highly expressed chloroplast transcripts were selected, which account for approximately 55% of the total amount of chloroplast RNAs. Next, we made biotinylated anti-chloroplast RNA probes through *in vitro* transcription with biotin-UTP. Denatured RNA gel images showed the high purity of these home-made biotinylated RNA probes (Figure 4B). We annealed the biotinylated probes on DMS-treated total RNA and depleted the targeted chloroplast transcripts with streptavidin magnetic beads (Figure 4A, see section “Materials and Methods”). RT-PCR results showed that, compared with the no-probe control, the amount of targeted chloroplast transcripts was decreased in probe-treated samples, suggesting that our depletion method was successful (Figure 4C). The result also showed a dosage-dependent effect of antisense probes on RNA depletion (Figure 4C). In addition, we observed the depletion efficiency of *PSBC* and *PSAB* is lower than other targeted chloroplast transcripts, indicating insufficient annealing between probes and targets (Figure 4C). It might contribute from DMS-induced modifications at the Watson-Crick face of A and C that compromise the probe-target annealing, or from the strong intramolecular RNA structure in targets that impedes probe interaction.

Notably, this streptavidin-biotin depletion approach could be applied to reduce tissue-specific highly-expressed transcripts in different tissue samples, increasing the sequencing depth and coverage of genome-wide DMS-MaPseq.

### Application of Genome-Wide DMS-MaPseq on Rice Materials

To apply genome-wide DMS-MaPseq on rice materials, we chose total RNAs from untreated and 1% DMS-treated leaf samples to construct genome-wide DMS-MaPseq libraries. rRNAs were depleted with Ribo-Zero Kit and the amount of chloroplast transcripts were reduced by the streptavidin-biotin depletion approach discussed above. Then, following a previously published protocol for Arabidopsis materials (Wang et al., 2019), we prepared the libraries for rice using a commercial RNA-seq library kit with some modifications in the RT step to be compatible with TGIRT (Figure 1). We generated one biological repeat for untreated and three biological repeats for DMS-treated samples. After sequencing and quality filtering, we mapped clean reads to the *Oryza sativa* L. ssp. Nipponbare reference genome (MSU Rice Genome Annotation Project Release 7) by TopHat with 10% mismatch tolerance (Zubradt et al., 2017). For both untreated and treated samples, most reads were mapped to the reference genome.

**Figure 5:**
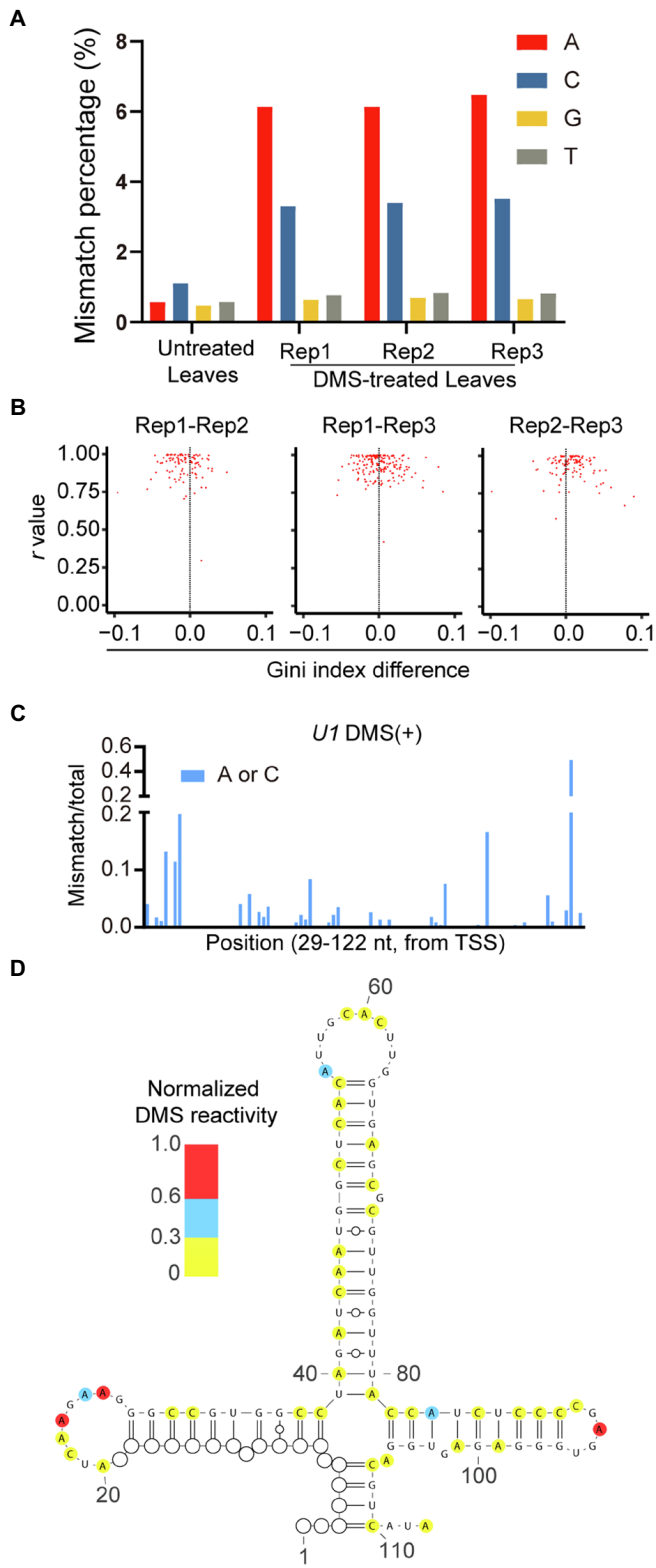
High-quality of genome-wide DMS-MaPseq data in rice. **(A)** Total mismatch percentage on each nucleotide in untreated and DMS-treated samples. **(B)** Reproducibility between DMS-treated biological repeats were measured by Pearson’s *r* value and Gini index. **(C)** Ratiometric DMS signals of each A and C were plotted along *U1* snRNA sequence. **(D)** The RNA secondary structure of 4-way junction region of *U1* snRNA was predicted based on DMS-MaPseq data. The ratiometric DMS signal per position normalized to the highest mismatch/total in the displayed region, which was set arbitrarily to 1.0. White cycles indicated nucleotides without read coverage.

Next, we assessed the quality of our genome-wide DMS-MaPseq data. Compared with the untreated sample, the increased mismatches were specific to A and C in DMS-treated samples (Figure 5A). This is consistent with the mode of DMS modification. Moreover, the mismatch ratio of A was slightly higher than that of C, similar to the published results in human, yeast, and Arabidopsis (Zubradt et al., 2017; Wang et al., 2019). Next, we used Pearson’s *r* value and Gini index to measure the reproducibility among three DMS-treated biological replicates and obtained a high *r* value and a small Gini index difference among repeats, indicating the strong reproducibility of our data (Figure 5B). Therefore, we merged the DMS-MaPseq data of the three DMS-treated biological replicates for further analysis.

To validate the feasibility of our genome-wide DMS-MaPseq data for *in vivo* RNA secondary structure prediction, we used our DMS mutation signals as constraints to predict the *in vivo* RNA secondary structure of *U1* small nuclear RNA (snRNA) with known RNA structure. The predicted RNA secondary structure exhibited a four-way junction, highly consistent with the reference structure (Krummel et al., 2011; Figures 5C,D). Altogether, these results demonstrated that genome-wide DMS-MaPseq could be applied to rice materials and produce high-quality *in vivo* RNA structure information.

### *In vivo* RNA Secondary Structure Modeling

We applied our optimized DMS-MaPseq to investigate the regulatory functions of *in vivo* RNA secondary structure in rice. miRNA-mediated cleavage participates in various aspects of developmental and stress responses by suppressing gene expression or translation in plants (Rogers and Chen, 2013; Li et al., 2017). miR399 was the first identified miRNA involved in stress responses in plants (Fujii et al., 2005; Bari et al., 2006; Chiou et al., 2006). It is a key regulator of inorganic phosphate (Pi) homeostasis and the phosphate-starvation response pathway (Fujii et al., 2005; Bari et al., 2006; Chiou et al., 2006). miR399 binds to the 5′UTR of *PHO2* and reduces its expression through post-transcriptional miRNA-mediated cleavage of the *PHO2* mRNA (Lin et al., 2008; Pant et al., 2008). *PHO2* encodes a ubiquitin-conjugating E2 enzyme, that participates in protein degradation of the Pi exporter PHO1 (Liu et al., 2012). The effect of mRNA structure on miRNA-mediated cleavage has been studied in human, *C. elegant*, *Drosophila*, and Arabidopsis (Ameres et al., 2007; Long et al., 2007; Yang et al., 2020a), but it is unclear how mRNA structure regulates miR399-mediated cleavage.

**Figure 6:**
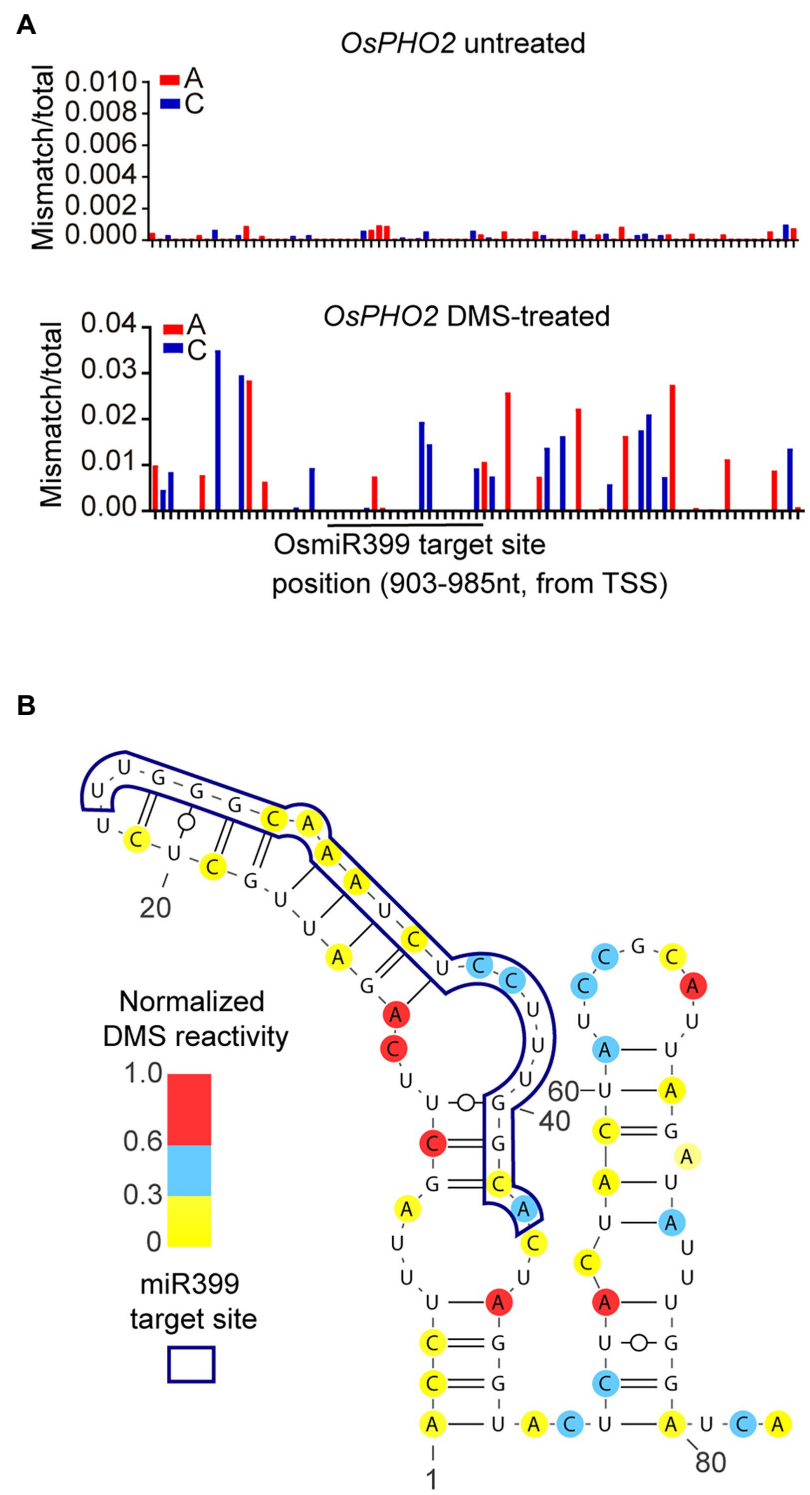
Profiling RNA secondary structure of a OsmiR399 target site located at 5′UTR of *OsPHO2*. **(A)** Ratiometric DMS signals of each A and C were plotted along the tested 5′UTR of *OsPHO2*. The OsmiR399 target site was shown by a black line. **(B)** The RNA secondary structure of an OsmiR399 target site located at 5′UTR of *OsPHO2* was predicted based on our DMS-MaPseq data. The ratiometric DMS signal per position normalized to the highest mismatch/total in the displayed region, which was set arbitrarily to 1.0.

Detecting the effect of mRNA structure on miR399-mediated cleavage requires probing *in vivo* RNA structure of *PHO2* before cleavage. Regular genome-wide RNA structure probing methods only provide population-average RNA structure information, resulting in that RNA structures of pre-cleaved and cleaved miRNA-target mRNAs are indistinguishable. However, target-specific DMS-MaPseq can probe isoform-specific RNA secondary structures (Zubradt et al., 2017). Therefore, we used target-specific DMS-MaPseq to profile the *in vivo* RNA secondary structure of the OsmiR399 target site and its flanking region of pre-cleaved *OsPHO2* transcripts in rice. The DMS-MaPseq data showed no obvious difference in DMS mutation signals between the miRNA target site and its flanking region, consistent with a previous finding that miRNA target sites are not structurally accessible for binding the miRNA-induced silencing complex (Yang et al., 2020a; Figure 6A).

Next, we modeled the *in vivo* RNA secondary structure of this region based on our DMS-MaPseq data and observed a single-stranded structure immediately downstream of the miRNA target site (Figure 6B). Our predicted *in vivo* RNA secondary structure supported a published conclusion that the single-stranded structure downstream of miRNA target sites, named Target Adjacent nucleotide Motif (TAM), facilitates miRNA cleavage (Yang et al., 2020a). Our results suggested a regulatory function of mRNA structure on miR399-mediated Pi homeostasis, and also validated that our optimized DMS-MaPseq method could be used to investigate the biological function of *in vivo* RNA secondary structure in rice.

## Discussion

RNA structure is considered to be another layer of gene expression regulation, participating in various aspects of RNA metabolism (Bevilacqua et al., 2016; Vandivier et al., 2016; Yang et al., 2018; Zhu et al., 2021). However, compared to model species, studies of *in vivo* RNA structure-dependent biological functions in crops are rare (Deng et al., 2018; Yang et al., 2021).

There are some hurdles for RNA structure study in crops. First, the complexity of the cell wall and presence of multiple cell layers hinder penetration of small chemicals into cells to react with RNA. For instance, rice deposits silica bodies on the leaf and stem surface to resist pathogen infection and abiotic stress (Ma and Yamaji, 2006), which also block penetration of small chemicals, thereby hindering chemical-modification-based *in vivo* RNA structure probing. Furthermore, rice roots contain multiple layers of cortex cells (from several to more than 10 layers), while Arabidopsis roots contain only one cortical-cell layer (Henry et al., 2017). Multiple cell layers hinder chemical uptake in inner cells. To deal with this issue, we tested several DMS treatment conditions, including varying DMS concentrations and incubation conditions. We balanced the DMS-induced mutation ratio and RNA decay, and suggested that 1–2% DMS is suitable for rice leaf samples and 1% DMS is suitable for rice root samples (Figure 2). Our DMS treatment optimization for rice could serve as a reference for other crops, such as wheat (*Triticum aestivum*) and maize (*Zea mays*).

Secondly, due to their large genome size, crops require substantially more sequencing reads than Arabidopsis to achieve sufficient sequence depth and coverage for reliable genome-wide RNA structure information. The genome size of rice is relatively small compared to other major cereal crops, but it is still approximately threefold larger than the Arabidopsis genome (Yu et al., 2002). The level of coverage needed for the DMS-MaPseq method, such as 20× mismatch coverage, dramatically increases sequencing costs. In addition, low-abundance transcripts are difficult to detect. To solve this problem, some studies used etiolated plants and mRNA enrichment to reduce the amount of highly-expressed chloroplast RNAs (Deng et al., 2018; Su et al., 2018). The downsides of this are that etiolated plants are under stress condition, and mRNA enrichment could miss RNAs lacking a poly(A) tail. Alternatively, we used a streptavidin-biotin depletion approach to specifically reduce the abundance of highly-expressed chloroplast transcripts from total RNA, allowing for greater sequencing depth and coverage of low-expressed transcripts (Figure 4). Another strategy is using the target-specific DMS-MaPseq method to specifically profile the RNA secondary structure of low-expressed genes. It is worth noting that target-specific DMS-MaPseq can also detect *in vivo* RNA secondary structure from different isoforms (Figure 6; Zubradt et al., 2017).

The first *in vivo* RNA structurome of rice was profiled by the Structure-seq (Deng et al., 2018). Both Structure-seq and DMS-MaPseq use DMS modification for RNA structure probing. DMS modifications on RNA are decoded through RT mutation in our optimized DMS-MaPseq method, instead of RT stop in Structure-seq. DMS-MaPseq increases RNA structure information content in sequencing data and reduces false-positive signals from unwanted RNA decay (Wang et al., 2021). However, RT mutation-based DMS-MaPseq method requires a greater sequencing depth to generate accurate RNA structurome (Zubradt et al., 2017; Wang et al., 2019). Together, our optimized DMS-MaPseq is complementary to the Structure-seq.

Studies have shown that RNA structure plays important roles in abiotic stress responses in plants (Anderson et al., 2018; Su et al., 2018; Chung et al., 2020; Kramer et al., 2020; Tack et al., 2020; Reis et al., 2021). However, the biological functions of RNA structure in plant nutrient-deficiency stress are still elusive. Pi is an essential nutrient for crop growth and production (Oldroyd and Leyser, 2020). Due to the low solubility and slow diffusion of Pi in soil, approximately 70% of global cultivated land suffers from Pi deficiency (Raghothama, 1999; Lopez-Arredondo et al., 2014; Paz-Ares et al., 2022). To sustain modern agriculture and global crop yield, it is of great importance to understand plant Pi-starvation responses (PSR) and improve Pi utilization efficiency of crops. A recent report showed that the Pi-starvation induced long non-coding RNA *cis-NAT_PHO1;2_* enhances the translation of the Pi exporter gene *PHO1;2 via* an internal RNA–RNA interaction and RNA structure change (Reis et al., 2021). Here, we used target-specific DMS-MaPseq to profile the *in vivo* RNA secondary structure of an OsmiR399-target region of *OsPHO2*, which is a key PSR gene involved in Pi transport (Lin et al., 2008; Pant et al., 2008; Liu et al., 2012). We found a single-stranded region downstream of the miR399 target site (Figure 6), which may facilitate miRNA-mediated cleavage (Figure 6). These findings shed light on the regulatory function of RNA structure in plant nutrient metabolism. As *OsPHO2* contains five miR399 target sites, it would be interesting to investigate whether single-stranded RNA structure are exhibited in other miR399 target regions. Moreover, the miR399-*PHO2* regulatory mechanism is conserved across angiosperms (Bari et al., 2006), whether such RNA structure exists beyond rice also be an attractive topic for future study.

In this study, we presented an optimized and powerful DMS-MaPseq method for studying the biological functions of RNA structure in rice. We hope that this method, together with other advanced RNA structure probing approaches, will promote RNA structure-guided molecular breeding and crop improvement.

## Data Availability Statement

The datasets presented in this study can be found in online repositories. The names of the repository/repositories and accession number(s) can be found below: National Center for Biotechnology Information (NCBI) BioProject database under accession number GSE197245.

## Author Contributions

ZW conceived and designed research. QJ, SH, and GW conducted experiments. QJ and LZ performed data analysis. ZW and QJ wrote the manuscript. All authors contributed to the article and approved the submitted version.

## Funding

This work was supported by grants from the National Key Research and Development Program of China (2021YFF1000402) and the National Natural Science Foundation of China (32170262).

## Conflict of Interest

The authors declare that the research was conducted in the absence of any commercial or financial relationships that could be construed as a potential conflict of interest.

## Publisher’s Note

All claims expressed in this article are solely those of the authors and do not necessarily represent those of their affiliated organizations, or those of the publisher, the editors and the reviewers. Any product that may be evaluated in this article, or claim that may be made by its manufacturer, is not guaranteed or endorsed by the publisher.

## Footnotes

^1^ https://www.bioinformatics.babraham.ac.uk/projects/trim_galore/

^2^ https://ccb.jhu.edu/software/tophat/index.shtml

^3^ http://rna.urmc.rochester.edu/RNAstructure.html

^4^ http://varna.lri.fr/

^5^ https://www.graphpad.com/scientific-software/prism/

^6^ https://www.r-project.org/
